# Construction of Thick Myocardial Tissue through Layered Seeding in Multi-Layer Nanofiber Scaffolds

**DOI:** 10.3390/polym16182664

**Published:** 2024-09-22

**Authors:** Yuru You, Feng Xu, Lingling Liu, Songyue Chen, Zhengmao Ding, Daoheng Sun

**Affiliations:** Pen-Tung Sah Institute of Micro-Nano Science and Technology, Xiamen University, Xiamen 361000, China; 19920201151476@stu.xmu.edu.cn (Y.Y.); xu_feng_bp@aliyun.com (F.X.); l18850076019@163.com (L.L.); s.chen@xmu.edu.cn (S.C.)

**Keywords:** heart-on-a-chip, cell-layered seeding, thick myocardial tissue, anisotropic

## Abstract

A major challenge in myocardial tissue engineering is replicating the heart’s highly complex three-dimensional (3D) anisotropic structure. Heart-on-a-chip (HOC) is an emerging technology for constructing myocardial tissue in vitro in recent years, but most existing HOC systems face difficulties in constructing 3D myocardial tissue aligned with multiple cell layers. Electrospun nanofibers are commonly used as scaffolds for cell growth in myocardial tissue engineering, which can structurally simulate the extracellular matrix to induce the aligned growth of myocardial cells. Here, we developed an HOC that integrates multi-layered aligned polycaprolactone (PCL) nanofiber scaffolds inside microfluidic chips, and constructed 3D thick and aligned tissue with a layered seeding approach. By culturing human-induced pluripotent stem-cell-derived cardiomyocytes (hiPSC-CMs) on chip, the myocardial tissue on the two layered nanofibers reached a thickness of ~53 μm compared with ~19 μm for single-layered nanofibers. The obtained myocardial tissue presented well-aligned structures, with densely distributed α-actinin. By the third day post seeding, the hiPSC-CMs contract highly synchronously, with a contraction frequency of 18 times/min. The HOC with multi-layered biomimetic scaffolds provided a dynamic in vitro culture environment for hiPSC-CMs. Together with the layered cell-seeding process, the designed HOC promoted the formation of thick, well-aligned myocardial tissue.

## 1. Introduction

Engineered myocardial tissues have shown promising application in personalized heart disease research with the development of human-induced pluripotent stem cell (hiPSC) differentiation technology [1,2,3,4]. The typical human ventricular muscle comprises 3D quasi-layered tissue [5], and is characterized by a brick-like cell shape and highly organized sarcomeres [6], ensuring the electromechanical properties of the tissue. However, hiPSC-derived cardiomyocytes (hiPSC-CMs) grown on traditional planar substrates often exhibit random distribution, short sarcomeres, and underdeveloped excitation–contraction coupling [7]. Although 2D patterned micro-grooves [8,9] or nanofibers [10,11] provide constraints for ordered myocardial tissue formation, the inherent problem of 2D culture remains [5]. The 2D patterned structures only induce myocardial cells to form a monolayer of myocardial tissue on the substrate for cell growth, which is significantly different in the morphological dimension from natural myocardial 3D thick tissue. This immature tissue state failed to mimic the biological and physiological parameters of natural heart tissue [12,13]. Therefore, constructing myocardial tissue with a 3D topological morphology that mimics in vivo conditions is critically important for constructing myocardial tissue in vitro.

Currently, cell perfusion in scaffolds is the main method for culturing ordered, thick tissues [14,15]. The topological induction to cells was realized through structural design [16], e.g., 3D porous structures based on collagen and elastin sponges [17] or on silk fibroin and polypyrole sponges [18], and 3D-printed scaffolds based on fiber-infused gel [19] or by combining 3D-printed polymer and nanofibers [20]. Direct perfusion in 3D scaffolds provided 3D growth space for cells [21,22]. However, the current cell seeding method on 3D scaffolds faces problems such as the limited penetration ability of cells in the scaffold [23,24]. Although, the consistency of cell seeding can be improved by increasing pore size [23], enhancing perfusion pressure [25,26,27]. However, enlarged gaps can easily lead to isolated areas of cells, and excessive perfusion pressure can damage the viability of cells. Another solution is the layer-by-layer stacking of seeded 2D scaffolds [28,29]. Each layer has oriented cells cultured on 2D scaffolds; then, each multiple-cell layer is manually stacked to form thick tissues, which is a challenge due to the limited efficiency of manual stacking.

Here, we have designed an HOC, integrated with PDMS and PCL nanofibers for constructing thick anisotropic myocardial tissue by integrating multiple layers of aligned PCL nanofiber scaffolds within microfluidics. Electrospinning technology can produce thin membranes of a micro-nano scale, and with aligned and complex structures [30]. PCL is a widely used scaffold material due to its biocompatibility, cost effectiveness, thermal stability and mechanical properties [31,32]. The HOCs’ assembly adopts the oxygen plasma bonding method, which not only ensures the sealing of the HOCs but also improves the hydrophilicity of PCL nanofiber membranes, which is conducive to cell adhesion [33]. A layered seeding method has been developed for the HOC with multi-layer nanofiber scaffolds. Compared with the top-down seeding method, the layered seeding provides a thicker tissue formation. By culturing hiPSC-CMs on the nanofiber scaffolds inside the HOCs, the myocardial tissue reached a thickness of ~53 μm for the double-layered nanofibers, compared with ~19 μm for single-layered nanofibers. By increasing the nanofiber layers, the thickness of the tissue can be further improved. The 3D myocardial tissue with a certain thickness within the HOC expressed densely distributed α-actinin and highly synchronous contractions, with a frequency of 18 times/min.

## 2. Materials and Methods

### 2.1. Preparation and Characterization of PCL Nanofibers

PCL nanofiber scaffolds were fabricated via electrostatic spinning. PCL (Mn = 80,000, Sigma-Aldrich, St. Louis, MO, USA) was dissolved in acetic acid (analytically pure AR, LUYIN, Xiamen, China), and the solution was configured at a concentration of 20%, the spinning environment was set to a temperature of 25 °C and a humidity of 50%. A precision syringe pump (D300401, Harvard Apparatus, Holliston, MA, USA) was used to control the solution feed and set the solution feed volume to 0.5 mL/h. The high-voltage power supply (DW-P503-1ACD1, Dongwen High Voltage Power Supply, Tianjin, China) controlled the electrospinning voltage and set the spinning voltage to 8.5 kV. The drum-spinning method was employed to produce aligned PCL nanofibers. The collection device was affixed to a rotating drum at a speed of 1500 rpm, and the injector nozzle was placed perpendicular to the drum. The distance from the outlet to the collection device was set at 5 cm, and the spinning time was 10 min. For producing random nanofibers, the collection device was positioned on a flat surface and the injector nozzle was arranged perpendicular to the collection. The distance from the outlet to the collection device was set to 15 cm, and the spinning time was 10 min.

Scanning electron microscopy (SEM, JEOL, Tokyo, Japan) was employed to image the surface and lateral section structural morphology of the nanofiber scaffolds. Prior to observation, all PCL nanofibers underwent a two-minute sputter coating with a gold layer. The nanofiber diameter distribution, nanofiber density, and porosity were counted separately using ImageJ1 and subjected to graphical analysis using Origin2021 software.

### 2.2. Chip Design and Fabrication

The HOC had 2 × 2 arrays of functional structures, with a length of 47 mm, a width of 25 mm, and a height of 6.5 mm, assembled with a top layer, two nanofiber scaffold layers, and a bottom layer. Chambers and channels were designed within the top and bottom layers for cell seeding and culture medium infusion. Two PCL nanofiber scaffolds were employed as substrates for cellular growth, integrated within the top and bottom layers. The chambers of the top and bottom layers were enclosed to form culture areas (3000 µm × 5000 µm × 4000 µm); the medium inlet channel on the top layer is 800 µm × 2000 µm, the cell seeding channel is 800 µm × 150 µm, and the medium inlet channel on the bottom layer is 1000 µm × 500 µm.

PS fluorescent microspheres (6-1-1500, BaseLine ChromTech Research Centre, Tinajin, China) with a diameter of 15 μm were used to simulate the cells, which were seeded onto the single-layer and the multilayer nanofiber scaffolds with the top-down static seeding method and the layered seeding method, respectively. After 4 h of seeding, the distribution of PS fluorescent microspheres on the nanofiber scaffolds under different seeding methods was observed using a confocal laser microscope (FV3000, OLYMPUS, Tokyo, Japan). The relative positions of the fluorescent microspheres on the different layers of the multilayer nanofiber scaffolds were observed to determine the suitable spacing between layers.

**Figure 1:**
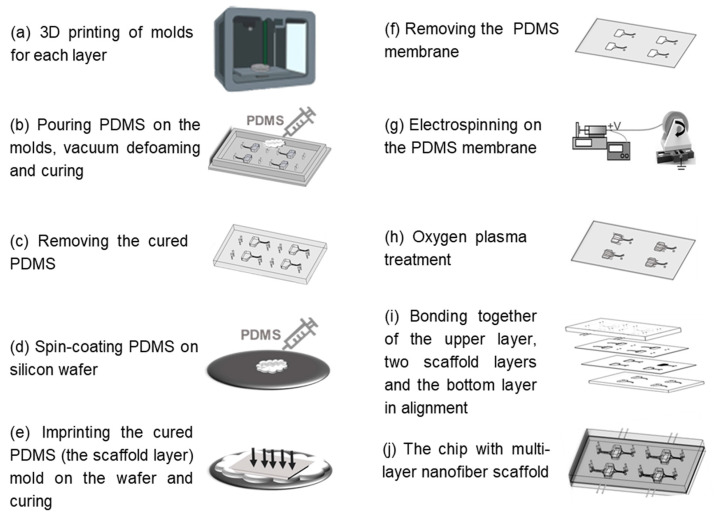
Schematic of fabrication steps of the HOC.

The HOC preparation process is shown in Figure 1, and the molds of each layer were prepared by a light-curing 3D printer (S240, BMF PRECISION TECH, Shenzhen, China) with a printing accuracy of 10 μm. The top and bottom layers of the chip can be obtained directly by pouring PDMS into the molds, heating and curing it, and then demolding it. The preparation of the PCL nanofiber scaffold layers requires the following: 1. Pouring PDMS into the 3D-printed scaffold layer mold for curing and demolding to obtain the scaffold layer mold (PDMS); 2. Spin-coating a layer of PDMS onto the wafer, inverting the scaffold layer mold (PDMS) on the wafer, and obtaining the scaffold layer’s PDMS membrane by heating and curing; 3. Fixing the PDMS film on a roller for electrostatic spinning and then performing electrospinning. Finally, the surfaces of the top layer, the two nanofiber scaffold layers, and the bottom layer were sequentially treated with oxygen plasma (PLUTO-T, BasalMedia, Shanghai, China) at 100 W power for 90 s, and then the layers of the chip were bonded layer by layer within 1 min. All steps pertaining to the curing of PDMS in the preparation process were vacuumed under a negative pressure of 0.5 MPa for 20 min before curing to remove air bubbles, and then heated at 60 °C for 30 min to complete the curing process.

### 2.3. Chip Performance Test and Characterization

The bonding strength of the chip was tested using a combination of liquid and gas pressure. The specific steps were as follows: first, liquid was perfused into the chip with integrated PCL nanofiber scaffolds, and then PDMS was poured into three of the infusion channels’ entrances to seal the entrances. After the PDMS was cured at room temperature, compressed gas was injected into the only remaining infusion channel entrance of the chip by the microfluidic pressure pump (Flow EZ, Fluigent, Paris, France), and the pressure value was continuously increased to observe in real time whether there was solution leakage from the chip. The microfluidic pressure pump was connected to a flow sensor (Flow Unit, Fluigent, Paris, France) for real-time testing of the inlet pressure values at different outlet flow rates.

### 2.4. Cell Culture and Characterization

Before cell seeding, all culture devices were sterilized by gamma rays (30 kgy) for 5 min. The culture devices were placed in an incubator (37 °C, 5% CO_2_) to be coated with 0.15 wt.% pig gelatin (V900863, Sigma) for 24 h.

HiPSC-CMs transfected with green fluorescent protein (GFP) (Beating Origin, Foshan, China) were grown in RPMI 1640 medium supplemented with 3% KnockOut serum substitute (Gibco, New York, NY, USA). All cells were maintained in an incubator, with 37 °C and 5% CO_2_. Before seeding the cells, we first digested them from the Petri dish with 0.05% trypsin (Gibco, New York, NY, USA). The seeding concentration of cells into the HOC was 5 × 10^6^ cells/mL. The cells were layered when seeding into the HOC, and we placed them in an incubator for 4 h. After the cells attached, we used a pressure pump (ELUPPU1000PCK, Fluigent, Paris, France) to continuously inject the culture medium into the chip at a flow rate of 120 μL/min.

For immunofluorescence staining, cardiomyocytes were fixed with 4% paraformal-dehyde for 20 min, followed by permeabilization with 0.25% Triton-X 100 (Sigma-Aldrich, St. Louis, MO, USA) in Phosphate-buffered saline (PBS) for 20 min at room temperature. After incubating in the blocking buffer (10% goat serum in PBS), cells were stained with primary antibodies (Monoclonal Anti-α-Actinin (Sarcomeric), A7811, Sigma-Aldrich, St. Louis, MO, USA) at 4 °C overnight. Cells were washed three times with PBS containing 0.1% Triton X-100, then fluorescent secondary antibodies (Alexa Fluor555 goat anti-rabbit, Az1428, Invitrogen, Carlsbad, CA, USA) were added for 60 min in the dark at 37 °C. The nuclei were labeled with Hoechst 33342 (C1022, Beyotime, Shanghai, China) for 10 min. The samples were washed with PBS at least 3 times between each step and maintained at 4 °C in PBS until image acquisition.

Optical methods were used for the automatic analysis of fluorescence images to determine cell contraction frequency [34]. A video of hiPSC-CMs spontaneously contracting was captured using an inverted fluorescence microscope (MF-52N, Mshot, Guangzhou, China). The video was imported into ImageJ and segmented into a series of single-frame images. The dwell time of each frame of the image can be calculated based on the video duration and image frame number. The fluorescence intensity of each single-frame image was detected to obtain a green fluorescence intensity waveform that changes over time. By measuring the distance between each peak of light intensity and taking the average value, the average contraction frequency of myocardial tissues can be calculated.

## 3. Results and Discussion

### 3.1. The HOC with Integrated the Multi-Layer Nanofiber Scaffold

**Figure 2:**
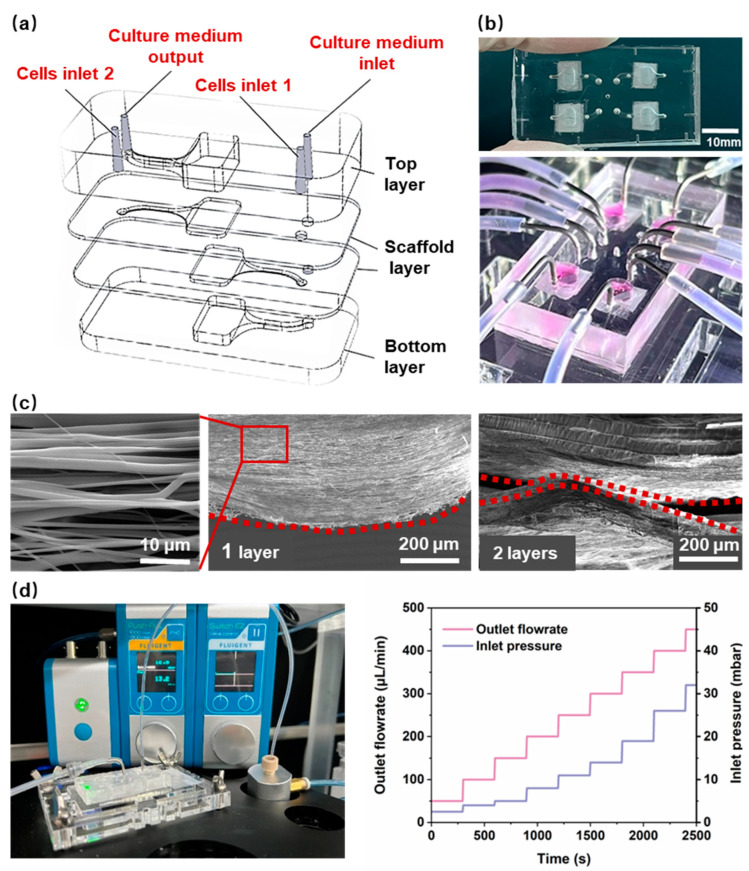
(**a**) The schematic diagram of the HOC structure. (**b**) An image of the HOC. (**c**) A scanning electron microscopy (SEM) image of a 1-layer and 2-layer aligned PCL nanofiber. The red dotted line highlights the side profile of the PCL nanofiber. (**d**) The bonding strength testing devices and the results of the HOC.

The design of the chip for 3D myocardial tissue was based on practical applications, with the objective of providing a biomimetic environment that is similar to the extracellular matrix for myocardial cells. This is intended to induce anisotropic cell growth and support continuous fluid stimulation for in vitro dynamic culture. An HOC, integrated with multilayers of PCL nanofiber scaffolds, was designed as shown in Figure 2a,b. The chip includes a top PDMS layer, two PCL nanofiber scaffold layers, and a bottom PDMS layer. Aligned nanofiber scaffolds were designed as cell growth substrates to induce cell alignment, simulating the anisotropic arrangement of natural myocardial tissue. The structure of stacking multiple layers of PCL nanofiber scaffolds can induce the growth of myocardial cells in terms of thickness. As a cell-growth substrate, PCL nanofiber scaffolds need to provide appropriate density and porosity to ensure cell growth and the formation of tissue. Given the size of myocardial cells (a diameter of 10–20 μm), the prepared PCL nanofibers (Figure 2c) have an average spacing of 5–15 μm, an average density of 1180 fibers/mm and a porosity of 36.1 ± 9.1%. Two nanofiber layers were integrated inside the chip, and the spacing between the nanofiber layers affects the formation of myocardial tissue. An interlayer spacing that is too small may not form a 3D thickness of myocardial tissue, while an interlayer spacing that is too large may prevent the cells on the two layers of nanofibers from contacting and interconnecting. Therefore, the spacing between the two layers of nanofibers was designed to be 300 μm. The real spacing is much smaller, as shown in Figure 2c, most probably due to the influence of gravity.

Anisotropic nanofiber scaffolds provide contact guidance for cells, thereby inducing a directional arrangement that results in higher aspect ratios and longer sarcomere lengths. This is an important theme for promoting the morphological and structural maturation of myocardial cells. At the same time, fluid stimulation can further promote the parallel arrangement, gene expression, and morphological maturation of myocardial cells, and induce stem-cell differentiation into myocardial cells. Therefore, the in vitro reconstruction of myocardial tissue not only requires a suitable 3D structure, but one also able to provide cells with a dynamic microenvironment [35]. To ensure that the nanofiber scaffold is not damaged during long-term medium infusion, the flow rate was set to 120 μL/min. The HOCs need sufficient bonding strength to ensure they remain leakage-free during the long-term continuous infusion of the culture medium. The PCL nanofiber scaffold in HOC is composed of nanofiber scaffolds and PDMS membranes. Consequently, the combination of nanofibers and PDMS represents a significant challenge in terms of sealing. We used the PDMS scaffold as the receiving substrate and directly electrospun nanofibers onto it. Before spinning, a mask was placed on the PDMS scaffold to restrict the attachment range of nanofibers and ensure the cleanliness of the remaining surface of the PDMS scaffold. Then, oxygen plasma was used to treat the surface of each layer (from bottom to top), followed by aligning and bonding the PDMS scaffolds layer by layer. Each layer surface must be aligned for bonding within 1 min after being treated with oxygen plasma. In order to facilitate chemical bonding between the PDMS layers after contact, heating was used to enhance the strength of the chemical bonding. The melting temperature of PCL nanofibers was 65 °C. Therefore, after bonding all layers, the HOC was subjected to 45 °C for 6 h to improve the bonding strength and ensure that the PCL nanofibers do not melt. The bonding strength of the HOC was ~300 mbar, above which value leakage occurred. Figure 2d shows the real-time measured air pressure values of pressure valves connected to flow sensors by the HOC at different flow rates. At a flow rate of 0–500 μL/min, the required air pressure value for the HOC was 0–50 mbar, which demonstrated that the bonding strength of the HOC met the flow rate requirements.

### 3.2. Comparison of Seeding Methods within the HOC

**Figure 3:**
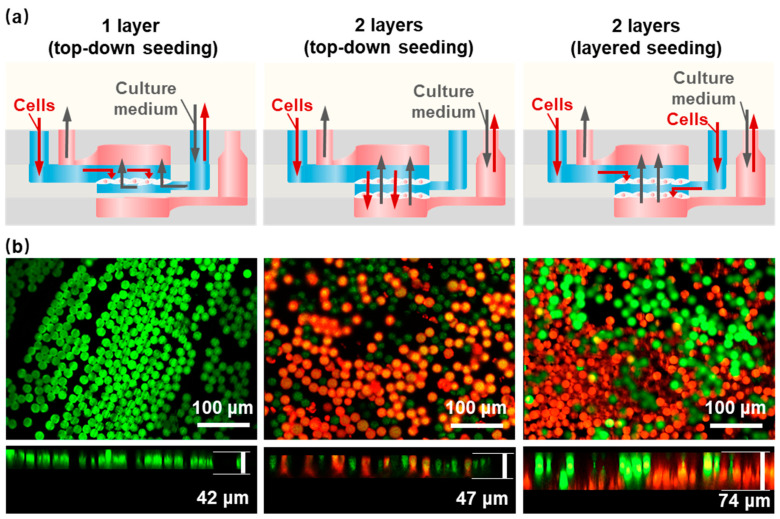
Different cell seeding methods within the HOC. (**a**) A schematic of different seeding methods. (**b**) PS microsphere fluorescence images of a 1-layer AF and a 2-layer AF with top-down and layered seeding methods. Green: the green fluorescent microspheres; Red: the red fluorescent microspheres.

The conventional approach to cell seeding is the top-down static seeding method, which is aimed at the seeding of single-layer scaffolds. We developed a cell-layered seeding method that is more suitable for HOCs containing multi-layer nanofiber scaffold structures to achieve the uniform diffusion of cells on each layer of the nanofiber scaffold. Figure 3a shows the comparison of cell seeding methods on different HOCs by seeding polystyrene (PS) fluorescent microspheres with either single-layer aligned nanofiber scaffolds (1-layer AF) and double-layer aligned nanofiber scaffolds (2-layer AF). After seeding the PS microspheres, the seeding thickness is ~42 μm for single-layer nanofiber, which did not improve much for the top-down seeding on 2-layer AF, with a thickness of ~47 μm (Figure 3b). In the latter case, the obstruction of the first layer of nanofibers resulted in the PS microspheres being concentrated on the top layer, thereby preventing contact with the second layer of nanofibers. If the porosity of the top-layer nanofibers is increased to facilitate the entry of PS microspheres into the second-layer nanofibers, it will make it difficult for PS microspheres to stay in the top layer, and most of them will fall onto the bottom-layer nanofibers, resulting in the uneven seeding density of different layers and affecting the formation of 3D thick tissue. In comparison, the layered seeding method, where two separate cell-seeding channels were designed for each nanofiber layer, demonstrated a nearly double thickness of ~74 μm. The confocal microscope images show the distribution and thickness of the seeding method, where the green fluorescent microspheres were seeded on the top layer and the red fluorescent microspheres were seeded on the bottom layer, with interconnected morphology. Therefore, we believe that the layered seeding method provided a more uniform distribution of microspheres in the vertical dimension, which was beneficial for the formation of 3D thick tissue. The culture medium adopted a bottom-up injection mode, which could ensure the sufficient exchange of the culture medium inside the culture reservoir.

### 3.3. Myocardial Tissue Formed within the HOCs

**Figure 4:**
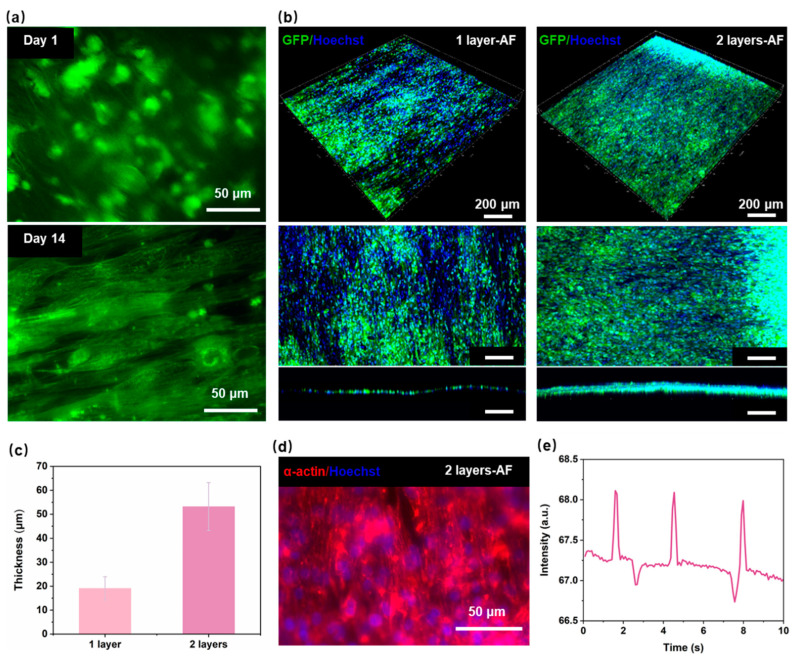
Performance of myocardial tissue on the HOC. (**a**) Fluorescence image of PCL nanofibers with hiPSC-CMs on day 1 and day 14. (**b**) Fluorescence image of the myocardial tissue on 1-layer AF and 2-layer AF. (**c**) The thickness of the myocardial tissue on 1-layer AF and 2-layer AF. (**d**) Fluorescence image of the α-actinin on 2-layer AF. (**e**) The contraction frequency of the myocardial tissue on 2-layer AF.

Figure 4a illustrate the seeding of hiPSC-CMs on the nanofiber on day 1 and day 14. On the initial day, the cells were observed to be randomly distributed on the nanofibers, and no evidence was found to suggest that the nanofibers were inducing the cells. Most of the cell-growth directions were not consistent with the arrangement direction of the nanofibers. Compared with the cells on day 1, the cells on day 14 on the aligned nanofibers extended along the nanofiber direction, showing a clear spindle-like shape. This indicates that the aligned nanofibers have a significant aligned induction effect on hiPSC-CMs. The main goal of assembling multiple layers of the PCL nanofiber scaffolds layer-by-layer within the HOC is to create myocardial tissue with a certain thickness and high anisotropy. To investigate the effect of multi-layer aligned nanofiber scaffolds on myocardial tissue thickness, we prepared different HOCs containing either one or two layers of AF for cell experiments. Figure 3b and Figure 4b shows that the thickness of myocardial tissue on the 2-layer AF is significantly higher than that on the 1-layer AF, with tight cellular connections observed between different layers on the 2-layer AF. Specifically, myocardial tissue thickness was measured as 19.1 ± 4.9 μm on 1-layer AF and 53.2 ± 10 μm on 2-layer AF (Figure 4c). The thickness of myocardial tissue formed by hiPSC-CMs on nanofibers thickens with the increase in the number of the nanofiber scaffold layers. This demonstrates that the thickness of the integrated tissue structure can be quantitatively adjusted by varying the number of nanofiber scaffolds.

One of the main goals of reconstructing myocardial tissue in vitro is to reproduce the contraction behavior of natural myocardial tissue in vivo [36,37,38]. The growth status of cardiac sarcomere and the protein expression of myocardial tissue are key markers of strong contractility and maturity in hiPSC-CMs; the content of α-actinin protein is closely related to myocardial tissue contractility [39]. Figure 4d shows that after 7 days of seeding, myocardial tissue can express good α-actinin on HOC with the 2-layer AF. By the third day post seeding, myocardial tissue demonstrates spontaneous contraction on the 2-layer AF (Figure 4e). The hiPSC-CMs on a 2-layer AF (3D myocardial tissue) contract highly synchronously, with a contraction frequency of 18 times/min. These indicate that our developed HOC with multi-layer PCL nanofiber scaffolds can provide a suitable in vitro microenvironment for the growth of myocardial cells, inducing myocardial tissue maturation and highly synchronized contraction behavior.

## 4. Conclusions

In this work, we developed an HOC that integrated multi-layer PCL nanofiber scaffolds, and realized cell seeding with a layered seeding method. This method improved the seeding thickness, as well as effectively induced hiPSC-CMs to form multi-layer aligned 3D myocardial tissue, facilitating interlayer cell communication and increasing tissue thickness. The thickness of myocardial tissue is increased with the layer number of nanofiber scaffolds, 19.1 ± 4.9 μm for 1-layer AF and 53.2 ± 10 μm for 2-layer AF. The different thicknesses of myocardial tissue can be obtained by adjusting the number of nanofiber scaffold layers according to different application scenarios. The hiPSC-CMs on the HOC expressed densely distributed α-actinin and produced highly synchronous contractions at a frequency of 18 times/min. These demonstrate the HOC’s potential for applications in personalized disease modeling, drug development, physiological research, and other fields.

## Data Availability

The original contributions presented in the study are included in the article material, further inquiries can be directed to the corresponding authors.
